# MiR199b Suppresses Expression of Hypoxia-Inducible Factor 1α (HIF-1α) in Prostate Cancer Cells

**DOI:** 10.3390/ijms14048422

**Published:** 2013-04-17

**Authors:** Weiwei Shang, Xueqin Chen, Ling Nie, Miao Xu, Ni Chen, Hao Zeng, Qiao Zhou

**Affiliations:** 1 Laboratory of Pathology, State Key Laboratory of Biotherapy and Department of Pathology, West China Hospital, West China Medical School, Sichuan University, Chengdu 610041, China; E-Mails: shangweiwei@gmail.com (W.S.); cxq19761980@126.com (X.C.); lingling830209@163.com (L.N.); miao6362@163.com (M.X.); chenni1@163.com (N.C.); 2 Department of Urology, West China Hospital, Sichuan University, Chengdu 610041, China; E-Mail: Kucaizeng@163.com

**Keywords:** miR-199b, HIF-1α, prostate cancer

## Abstract

MicroRNAs (miRNAs) are a class of small noncoding RNAs that post-transcriptionally repress expression of target genes *via* imperfect base-pairing with the 3′-untranslated region (3′-UTR). The transcription factor hypoxia-inducible factor-1α (HIF-1α) plays important roles in physiology and pathology. Constitutive over-expression of HIF-1α is observed in many types of cancers including prostate carcinoma, but the mechanisms underlying this event remain largely unknown. Here we investigated the expression of miR199b and HIF-1α in normal prostate tissue, prostate cancer tissues and prostate carcinoma (PCa) cell lines LNCaP, PC-3 and DU145.We found that miR-199b expression level was decreased in prostate cancer while HIF-1α was significantly over-expressed. Furthermore, we postulated the posttranscriptional regulation of HIF-1α by miR199b through bioinformatics analysis, and herein we experimentally demonstrated that miR199b negatively regulated HIF-1α by targeting its 3′-untranslated region. Artificial over-expression of miR199b by using adenoviral vectors in prostate cancer PC-3 and DU145 cells significantly down-regulated HIF-1α, together with reduced cell growth and increased cell death.

## 1. Introduction

Prostate cancer is the most commonly diagnosed cancer in men in the United States and in the majority of European countries [1,2]. It progresses from a localized, androgen-dependent disease to an advanced, invasive and metastatic stage with the loss of androgen-dependent [3]. Despite significant improvement of hormonal, surgical, chemical and radiation therapies, there is still no effective cure for androgen-independent prostate cancer. Once the androgen-independent phase develops, prostate cancer is always fatal. For this reason, unconventional strategies targeting novel molecular mechanisms are eagerly required.

The transcription factor HIF-1α plays pivotal roles in cellular response to hypoxia as well as in pathologic processes including carcinogenesis [4,5]. Many genes have been identified as targets of HIF-1α including TNF-α, BCL-xL, and VEGF, which are involved in biological processes such as cell proliferation, survival, and angiogenesis [6–8]. Over-expression of HIF-1α is implicated in the pathogenesis of many cancers including prostate carcinoma [4,5,9], in which it is associated with advanced clinical stage and chemo-resistance [10]. In addition, the reduced HIF-1α levels were found to be followed by suppressed tumor growth, angiogenesis, and metastasis [11]. HIF-1α over-expression has been identified in both prostate cancer tissue and cell lines [9,12,13]. Although over-expression of HIF-1α could be hypoxia-dependent, human prostate cancer cells can express functional HIF-1α protein excessively under normoxic conditions, the mechanisms of which remain largely unknown [12].

MicroRNAs (miRNAs) are a class of small non-coding RNAs (approximately 22–25 nt long) capable of combining with target mRNA in 3′-UTR for either inhibition of translation or degradation of mRNA [14]. MiRNAs are atypically expressed in a wide variety of human cancers, and thought to play important roles in tumorigenesis [15,16]. Increasing evidence supports that miRNAs participate in the development, prognosis and chemo-resistance of the prostate cancer [17–19].

Here, we identified HIF-1α mRNA is a direct target of miR199b. In addition, artificial over-expression of miR199b in prostate cancer cells may significantly impair the HIF-1α expression under normoxic and hypoxia-mimicking conditions, together with reduced cell growth and increased cell apoptosis. Our findings will help to further understand the functions of miRNAs in cancer and suggest that miR-199b may be employed as therapeutic for prostate cancer.

## 2. Results and Discussion

### 2.1. Expression of miR199b and HIF-1α in Prostate Cancer

Growing evidence indicates that atypical expression of miRNAs contributes to tumorigenesis [20,21]. A recent study showed that approximately 50% of annotated human miRNAs were associated with cancer [22]. Therefore, miRNAs are increasingly viewed as both biomarkers and therapeutic target [23,24]. MiR199b is downregulated in various human tumor types, including cancers of the colon, liver, brain, urinary bladder, breast, endometrium, and thyroid [25–28]. In some tumors, downregulation of miR199b is correlated with tumor size, stage, proliferative activity, or poorer prognosis [29–31]. Decrease of miR199b is observed in pre-malignant tissue and carcinoma [26], whereas artificial overexpression of miR199b inhibits cell growth and tumor formation [20,27,28]. Over-expression of HIF-1α and its target genes has been observed in a variety of solid tumors, such as tumors of the brain, lung, breast and prostate [9,32–34].

**Figure 1:**
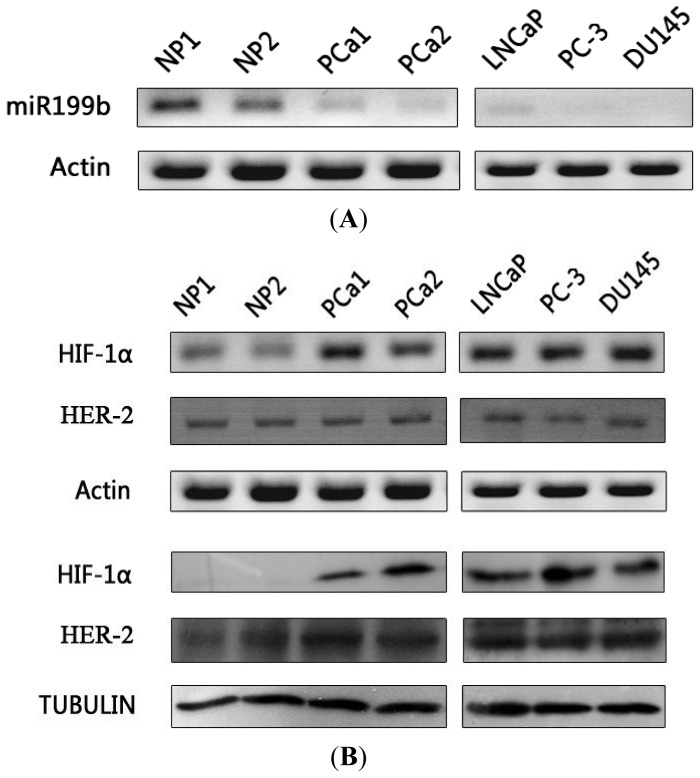
miR199b and HIF-1α expression in normal prostate (NP), prostate cancer tissue (PCa), and prostate cancer cell lines. (**A**) stem-loop RT-PCR analysis (with actin as control) showing differential expression of mature miR199b in normal prostate tissue *versus* prostate cancer, and prostate cancer cells LNCaP, PC-3, and DU145; (**B**) in contrast to miR199b, HIF-1α mRNA (top, RT-PCR, same actin control as for A) and protein (bottom, Western blotting, tubulin as control) were significantly higher in prostate cancer tissue and cells than in normal prostate tissue.

Here, expression of mature miR199b was investigated by stem loop RT-PCR, which showed very low expression level of miR199b in prostate cancer tissue and LNcaP cell in contrast with that in normal prostate tissue. In PC-3 and DU145 cells, no mature miR199b could be detected. Real-time PCR further showed that miR199b in prostate cancer tissue and LNcaP cell was only 1/38.7 and 1/69 respectively of that in normal prostate tissue, and was barely detectable in the other two prostate cancer cell lines (<1/10,000 of that in normal prostate tissue; data not shown). HIF-1α mRNA and protein overexpression in prostate cancer cell lines and primary prostate adenocarcinoma tissues was validated by conventional RT-PCR and Western blot analysis, respectively (Figure 1). In normal prostate tissue, HIF-1α mRNA and protein were undetectable. The expression pattern of miR199b is contrary to that of HIF-1α in normal prostate tissue and prostate cancer, and this situation prompt that HIF-1α may be regulated by miR199b negatively.

### 2.2. Identification of Potential HIF-1α 3′-UTR Seed Sequences

Identification of miRNA targets is one of the most important aspects in understanding the mechanisms by which miRNAs control cell behavior [35]. Several genes, including HER2, HES1, SET, PODXL and DDR1, have been identified as miR199b target genes [25,36,37]. HER2, a member of the epidermal growth factor receptor (EGFR/ErbB) family, which plays an important role in the pathogenesis and progression of certain aggressive types of breast cancer, is inhibited by miR199b, and artificial overexpression of miR-199b could suppress breast cancer cell proliferation and migration [25]. Inhibition of transcription factor HES1 by miR199b represses pluripotency in cancer stem cells and growth of medulloblastoma [38]. Moreover, miR-199b delivery through stable nucleic acid lipid particles in tumorigenic cells lines (colon, breast, prostate, glioblastoma and MB included) resulted in impairment of cell proliferation [39]. However, the roles of miR199b in carcinogenesis of many neoplasms are still to be elucidated.

In our study, the 1174 nt 3′-UTR of the HIF-1α mRNA (full-length 4082 nt, coding sequence 2504 nt) was analyzed by using Target- Scan 6.2 (http://www.targetscan.org/), which identified miR199b as the potential regulatory miRNA of HIF-1α. The 31 to 37 nt of the HIF-1α 3′-UTR was the potential seed sequence that was conserved across species. Sequence analysis showed no mutation or deletion of the 3′-UTR in PC-3, DU145, and LNCaP cells.

### 2.3. Dual Reporter Gene Assays Showed Interaction of miR199b with 3′-UTR of HIF-1α

**Figure 2:**
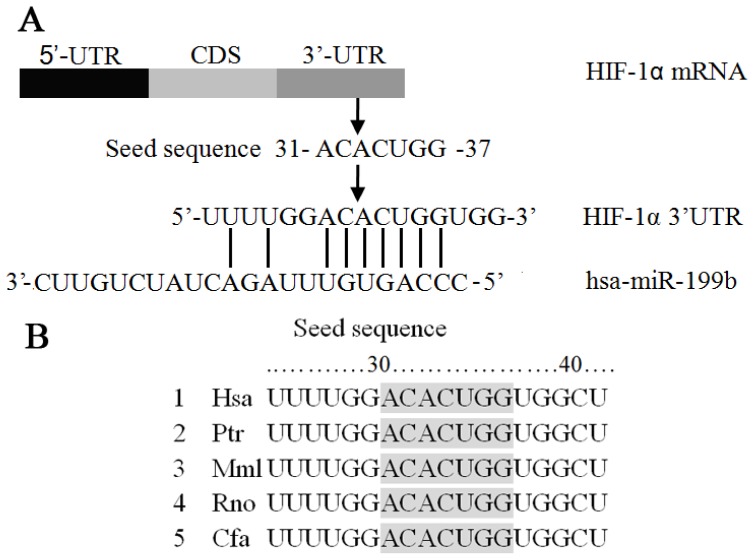

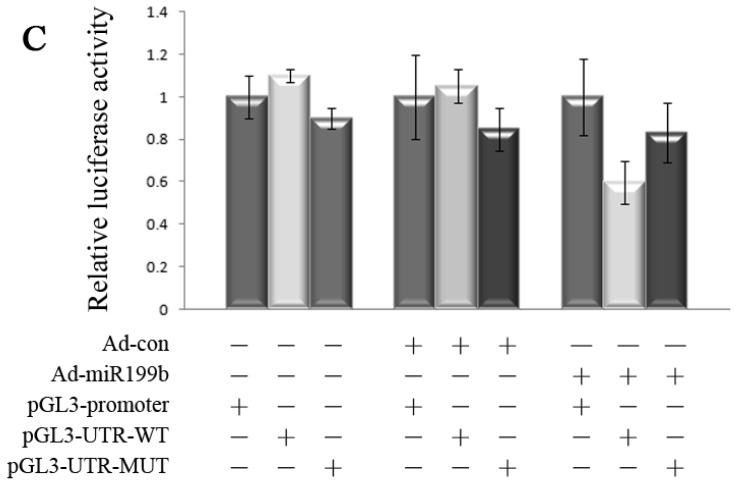
Identification of miR199b seed sequences in HIF-1α 3′-UTR and dual reporter gene assays for miR199b- HIF-1α 3′-UTR interaction. (**A**,**B**) The 31 to 37 nt of the HIF-1α 3′-UTR was identified as potential seed sequence for miR199b, designated as UTR-WT (**A**), which was conserved across species (**B**); (**C**) Dual reporter gene assays were performed with pGL3 expression constructs with HIF-1α 3′-UTR regions containing the seed sequences inserted downstream of the luciferase coding sequence, and the activity of the basic pGL3 construct as baseline (pGL3-Promoter). With the artificial expression of miR199b (by co-infection with AD-miR199b), the reporter gene activity, represented by relative luciferase activity (firefly/Renilla), was significantly decreased when UTR-WT was present in the constructs, whereas mutations of the seed sequences (UTR-MUT) significantly restored reporter gene activity. Expression of miR199b alone had no effect on reporter gene activity when no seed sequences were inserted.

To show posttranscriptional regulation of HIF-1α mRNA by miR199b, luciferase reporter gene constructs were prepared in which the potential seed sequence of HIF-1α 3′-UTR were cloned into luciferase reporter constructs, together with constructs in which the seed sequences were mutated. With artificial co-expression of miR199b by infection with Ad-miR199b, dual reporter assays showed significant down-regulation of luciferase reporter gene activity by 40% in the pGL3-UTR-wt constructs, whereas reporter constructs lacking HIF-1α 3′-UTR sequences were not affected. Moreover, mutation of the seed sequences significantly restored the luciferase gene activity in the constructs bearing the mutated 3′-UTR sequences (Figure 2C). Our study identified HIF-1α as a posttranscriptional target of miR199b. Because HIF-1α transcription could be regulated by NFκB [40], its overexpression in prostate cancer under normoxic conditions resulted from at least two fundamental molecular defects: overactivity of the transactivator NFκB at the transcriptional level and loss of the negative regulator miR199b at the posttranscriptional level.

### 2.4. Overexpression of miR-199b by Adenoviral Vectors Led to Down-Regulation of HIF-1α Protein, Inhibition of Cell Proliferation, and Increased Cell Death

**Figure 3:**
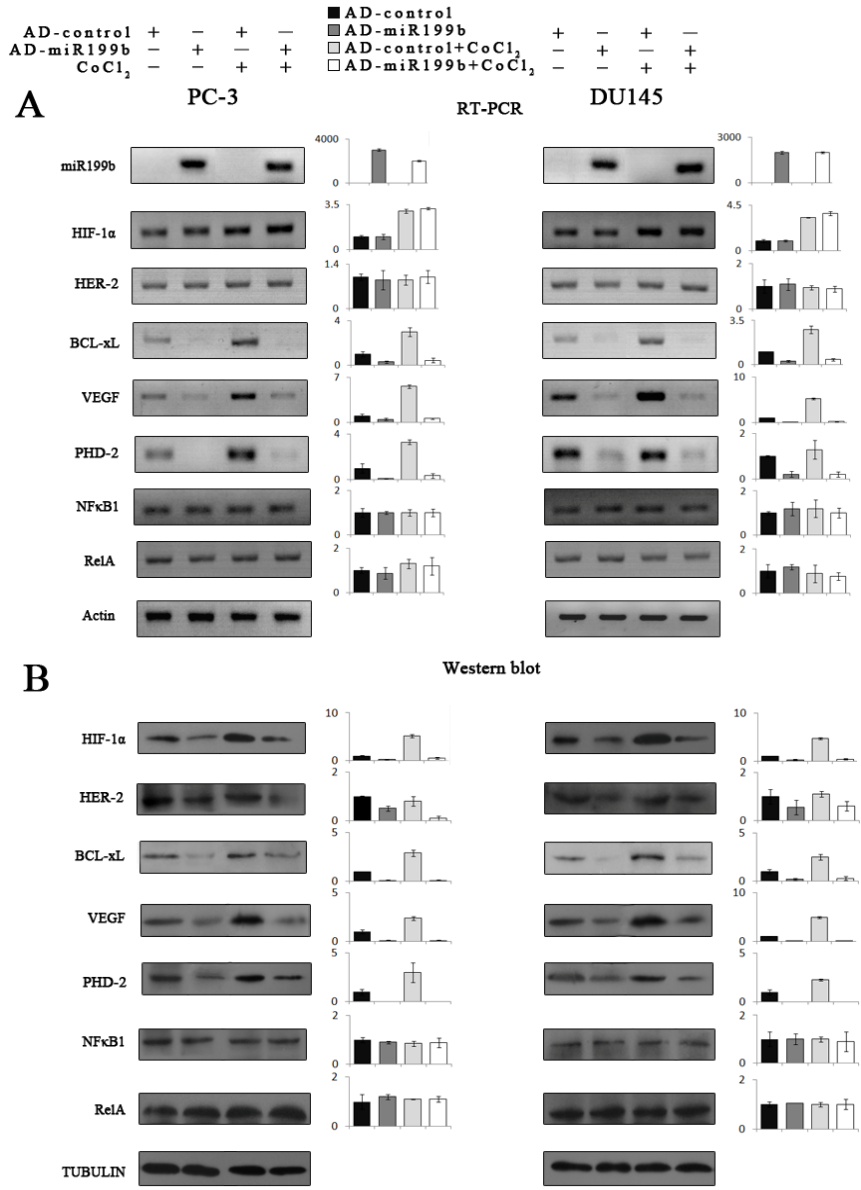
Effects on HIF-1α and its targets by artificial overexpression of miR199b in prostate cancer cells under normoxic and hypoxia-mimicking conditions. Artificial overexpression of miR199b by Ad-miR199b (**A**, top) resulted in the significant downregulation of HIF-1α protein level compared with Ad-control (**B**, Western blot with semiquantitative histograms) but no change in the HIF-1α mRNA or NFκB mRNA (**A**) or protein (**B**) levels (**A**, left, RT-PCR; right, Q-PCR). HIF-1α target genes BCL-xL, VEGF and PHD-2 were simultaneously deregulated upon miR199b overexpression (**A**) and HIF-1α downregulation (**B**).

HIF-1α upregulation contribute to resistance to radiotherapy and chemotherapy [4]. Knocking down of HIF-1α by siRNA could inhibit cell growth, proliferation, or migration, and promotes apoptosis in a variety of tumors [41,42]. There is an increased clinical interest in that silencing of HIF-1α gene results in sensitization of cancer cells to therapeutic agents. For example, knockdown of HIF-1α renders PC-3 cells more sensitive to UV or flutamide treatment, and this event supports that HIF-1α is a potential therapeutic target in androgen-independent prostate cancer [6]. These effects of inhibition are mainly mediated by suppression of HIF-1α targets involved in diverse physiological processes such as cell proliferation, metabolism, apoptosis and angiogenesis. These targets include phosphoglycerate kinase, GLUT-1, proly hydroxylases (PHD), anti-apoptotic factors survivin and BCL-xL, and VEGF [6,43–46]. The biological effects of HIF-1α regulation by miR-199b were further shown by assaying molecular and cellular changes in PC-3 and DU145 cells with artificial miR-199b over-expression. CoCl_2_ was used as a hypoxia-mimetic agent. Concomitant with the over-expression of mature miR-199b by Ad-miR-199b infection, HIF-1α protein level was significantly down-regulated under both normoxic and hypoxia-mimicking conditions. In contrast, the HIF-1α mRNA as well as NFκB (NFκB1, RelA) mRNA and protein showed little change with miR199b over-expression. We then examined the downstream target genes including Bcl-xl, VEGF and PHD-2, which are regulated by HIF-1α. Consistent with what we found, overexpression of miR199b suppressed the expression of these genes. Although the expression of HIF-1α and its target genes was boosted after CoCl_2_ treated, artificial overexpression of miR-199b could inhibit it effectively (Figure 3).

**Figure 4:**
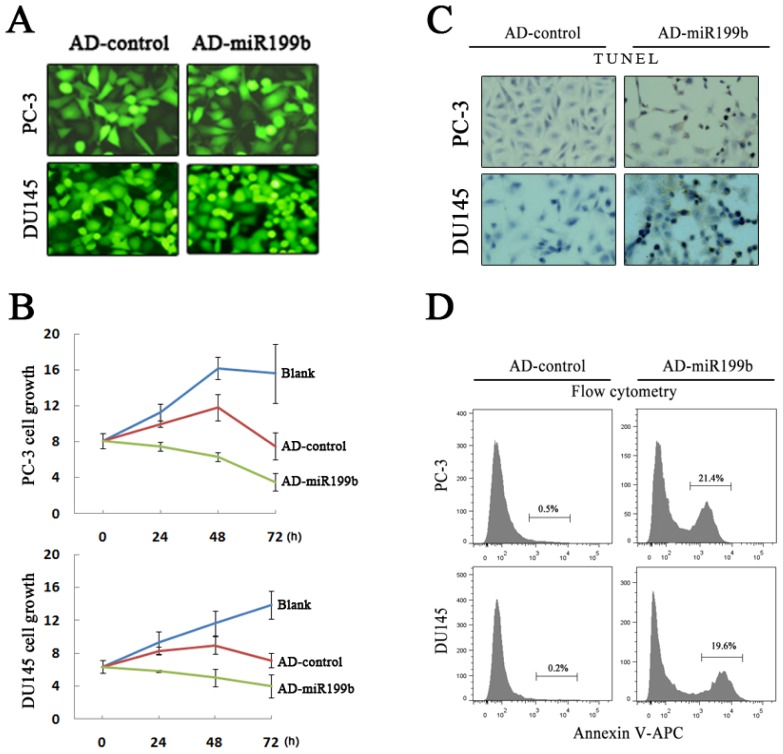
Cell behavior by artificial overexpression of miR199b in PC-3 and DU145 cells under normoxic condition. (**A**) the efficiency of Ad-miR199b and Ad-control infection was shown by homogenous green fluorescence protein expression of the infected cells; (**B**) cell growth was significantly inhibited concomitant with the miR199b- HIF-1α expression change; (**C**,**D**), increased cell death upon the miR199b- HIF-1α expression change as shown by flow cytometry analysis of percentage of Annexin V-APC–stained apoptotic cells (**D**) and TUNEL assays (**C**).

Synchronous with the HIF-1α expression change upon artificial miR199b over-expression, PC-3 and DU145 cells showed reduced cell growth and increased cell death (as assayed by TUNEL and FCM). Cellular viability and proliferation were measured following the protocol of the MTT assay kit at 24, 36, 48 and 72 h. Our results demonstrated that miR-199b could inhibit proliferation of prostate cancer cells obviously compared with Ad-control-treated or none-treated cultures. Suppression of cell growth by miR-199b was time-dependent. The apoptosis of PC-3 and DU145 cells was detected by TUNEL and FCM. Annexin V-APC/PI staining showed little to no detectable apoptosis in the Ad-control treated cells, while the percentage of apoptotic PC-3 and DU145 cells in the miR199b-treated cultures were 21.4% and 19.6%, respectively, significantly higher than the controls. TUNEL results also revealed the increased number of apoptotic prostate cancer cells after Ad-miR199b treated (Figure 4). Our data indicate that miR-199b can inhibit the proliferation and promote apototosis of the androgen-independent PC-3 and DU145 cells. In addition, the role of miR-199b in prostate cancer cell behaviors is mainly mediate by its function on HIF-1α target genes including BCL-xL, survivin and VEGF, *etc.*

### 2.5. Artificial Overexpression of miR-199b in HIF-1α Knockdown Cells

**Figure 5:**
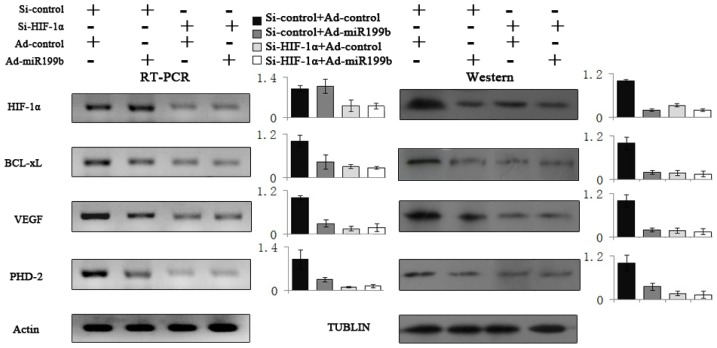
Effects on HIF-1α and its target genes by artificial over-expression of miR-199b in HIF-1α knockdown cells. Transfection of si-HIF-1α resulted in significantly decreased expression of HIF-1α and its target genes compared with si-control. Artificial overexpression of miR-199b by Ad-miR-199b could dramatically downregulate HIF-1α target genes expression in si-control treated PC-3 cells but fail to effectively inhibit these genes expression in si-HIF-1α treated cells. The HIF-1α mRNA expression wasn’t affected by Ad-miR-199b under any conditions of the above.

Since miR-199b can regulate multiple targets simultaneously and Bcl-xL, VEGF as well as PHD-2 may be regulated by other transcriptional factors besides HIF-1α, HIF-1α knockdown cells were constructed to confirm the specific effects of miR-199b on HIF-1α. Si- HIF-1α remarkably decreased expression of HIF-1α and its target genes, but the expression of these genes in si-control cells was not affected. Ad-miR-199b partially impaired HIF-1α expression but was unable to inhibit HIF-1α target genes effectively in si- HIF-1α treated cells. In contrast, artificial overexpression of miR-199b in si-control treated cells resulted in dramatically decreased HIF-1α protein as well as Bcl-xL, VEGF and PHD-2 expression (Figure 5). These results demonstrated that the effects of miR-199b on Bcl-xL, VEGF and PHD-2 were acted mainly through targeting HIF-1α directly.

## 3. Experimental Section

### 3.1. Cell lines and General Reagents

Human prostate cancer cells LNCaP, DU145, and PC-3 were from the American Type Culture Collection and were maintained in RPMI 1640 with 10% FCS (Life Technologies, Carlsbad, CA, USA). The adenovirusimmortalized human embryonic kidney epithelial cell HEK-293 was maintained in DMEM with 10% FCS. Tris base, Tween 20, DTT, and EDTA were from Amresco. Phenylmethylsulfonyl fluoride, leupeptin, pepstatin, and aprotinin were from Roche Diagnostics (Mannheim, Germany)

### 3.2. Stem-Loop Reverse Transcription and Conventional Reverse Transcription-PCR

Total RNA was extracted by using the Trizol reagent (Invitrogen, Carlsbad, CA, USA). The stem-loop reverse transcription-PCR (RT-PCR) technique was used to examine mature miR199b. The stem-loop RT primer was designed as miR199b (5′-GTC GTA TCC AGT GCA GGG TCC GAG GTA TTC GCA CTG GAT ACG ACG AAC AGA-3′). RT was carried out in 20 μL volume containing 2 μg of total RNA, 1.6 mmol/L miR stem-loop primer, 2 μL 10 mmol/L deoxynucleotide triphosphate, 1 μL 0.1 mol/L DTT, and 1 μL M-Mulv reverse transcriptase (Takara, Dalian, China) at 16 °C for 30 min, 42 °C for 30 min, 85 °C for 5 min and 10 °C for 3 min. The PCR primers for mature miR199b were designed as follows: sense, miR-199b (5′-GCC CGC CCA GTG TTT AGA CTA T-3′), anti-sense, 5′-GTG CAG GGT CCG AGG T-3′ product length, 66 bp). The random RT primer 5′-(dN)9-3′ (TaKaRa) was used for other genes, the PCR primers of which were designed according to their respective cDNA sequences (Genbank) as follows: HIF-1α (forward, 5′-CCT ATG ACC TGC TTG GTG CTG-3′, reverse, 5′-CTG GCT CAT ATC CCA TCA ATT CG-3′ product length, 157bp), HER-2 (forward, 5′-AGG GAA ACC TGG AAC TCA CC-3′, reverse, 5′-GCA CAA TCC GCA GCC TCT-3′ product length, 138bp) BCL-xL (forward, 5′-CTG TGC GTG GAA AGC GTA G-3′, reverse, 5′-CTC GGC TGC TGC ATT GTT C-3′ product length, 159bp), VEGF (forward, 5′-AGG AGG GCA GAA TCA TCA C-3′, reverse, 5′-AGG AGG GCA GAA TCA TCA CG-3′ product length, 140bp), PHD-2 (forward, 5′-ACC ATG AAC AAG CAC GGC ATC TGC-3′, reverse, 5′-GAC GTC TTT GCT GAC TGA ATT GGG CTT-3′ product length 215bp), NFκB1 (p105, forward, 5′-TGA ACT ACG AGG GAC CAG C-3′, reverse, 5′-TCA CTG GCT CTA AGG AAG G- 3′ product length, 358bp), RelA (p65 forward, 5′-AGC ACA GAT ACC CAC CAA GAC-3′, reverse, 5′-AGC ATT CAG GTC GTA GTC CC- 3′ product length, 318bp) and β-actin (forward, 5′-CTG GCA CCA CAC CTT CTA CAA TG-3′, reverse, 5′-CCT CGT AGA TGG GCA CAG TGT G-3′, 248 bp). Standard PCR protocols were used; products were resolved by 2% agarose gel or 15% PAGE and were visualized by staining with ethidium bromide or the fluorescent dye Goldview (SBS). Images were captured by scanning with Typhoon 8600 Multi-Imager (Molecular Dynamics, Sunnyvale, CA, USA) under fluorescence mode or with Bio-Rad Gel Doc XR (Bio-Rad, Hercules, CA, USA). Semiquantitative analysis was performed with the ImageQuant 5.2 software (Molecular Dynamics, Sunnyvale, CA, USA).

### 3.3. Real-Time Quantitative PCR

Real-time quantitative PCR (Q-PCR) was used together with stem-loop RT to quantitate mature miR199b. Q-PCR was performed on Light Cycler 2.0 (Roche), and data were analyzed with the Light Cycler software 4.05 (Roche) as described [6]. The β-actin gene was used as control. Copy number of target genes (relative to β-actin) was determined by the 2^−^_ΔΔCt_ method, with ΔΔCt = ΔCt_exp_ − ΔCt_con_ = (Ct_exp-target_ − Ct_exp-actin_) − (Ct_con-target_ −Ct_con-actin_), in which “exp” represents the experimental group, “con” the control group, and “target” the gene of interest.

### 3.4. Hypoxia Mimetic Treatment of PC-3 and DU145 Cells

PC-3 and DU145 cells were cultured in 6-well plates in FCS-free media, and treated with 400 μm of CoCl_2_ for 24 h. Cells were collected for Western and PCR.

### 3.5. Knockdown of HIF-1α Using Small Interfering RNA (siRNA)

HIF-1α knockdown was achieved using small interfering RNA (siRNAs; Dharmacon Inc), according to manufacturer’s instructions. PC-3 cells were cultured in 6-well plates in antibiotic-free media and transfected with si-RNAs in the presence of Lipofectamin 2000 (Invitrogen) for 24 h. None-specific siRNA was used as a negative control.

### 3.6. Western Blot

The primary antibodies used were as follows: HIF-α (rabbit polyclonal, 1:500, BD Biosciences Inc., Franklin Lakes, NJ, USA); HER-2 (1:500, Rabbit polyclonal, Boster, Wuhan, China); VEGF (1:500, Rabbit monoclonal, Boster, Wuhan, China); BCL-xL (rabbit polyclonal, 1:1000, Cell Signaling Technology Inc., Danvers, MA, USA); PHD-2 (1:1000, Rabbit monoclonal, Cell Signaling Technology Inc., Danvers, MA, USA); NFκB1 (p50, 1:500, Rabbit polyclonal, Boster, Wuhan, China); RelA (p65, 1:500, Rabbit polyclonal, Boster, Wuhan, China) and β-tubulin (mouse monoclonal, 1:1000, Huatesheng, Shenzhen, China). Horseradish peroxidase–labeled secondaryantibodies were from Zymed Laboratories, Inc. Nuclear extract preparation, total protein preparation and western blot analysis were carried out as previously described [6].

### 3.7. Recombinant Adenoviral Vectors for Overexpression of miR199b

The pri-miR199b sequence −267 to +295 (+1 being the first base of the mature miR199b) was amplified from HEK-293 cell genomic DNA with the primers described. PCR product was cloned into pMD18-T (TaKaRa), verified by sequencing, and subcloned into shuttle plasmid pAd-Track-CMV (designated as pAdTrack-miR199b). PAdTrack-miR199b linearized with PmeI was used to transform BJ5183-AD-1 cells harboring the adenoviral pAdeasy-1 vector (Stratagene) for homologous recombination. Colonies were screened by plasmid miniprep and PacI restriction analysis to obtain clones with recombinant miR199b (designated as pAdeasy-miR199b). PacI linearized pAdeasy-miR199b was used to transfect HEK-293 cells to obtain packaged recombinant miR199b adenovirus (designated as AD-miR199b). AD-miR199b was amplified by repeated infection and verified by PCR. The pAdTrack-CMV empty vector was used as control (designated as AD-control). The titers and the multiplicity of infection were determined according to the manufacturer’s protocols.

### 3.8. Cell Viability Assay

Cells were collected and stained with trypan blue (Sigma, 200 mg/mL, St. Louis, MO, USA). The number of viable cells was determined by microscopic examination.

### 3.9. MTT [3-(4,5-Dimethylthiazol-2-yl)-2,5-diphenyl-tetrazolium Bromide] Assay

The PC-3 and DU145 cells were placed in a 96 well plate (2 × 10^4^ cell/mL) overnight and then exposed to AD-miR199b. Cells incubated with AD-control served as control group. Cell viability was evaluated using the MTT assay 24, 48, 72 h respectively after incubation. In this assay, MTT is reduced to purple formazan in the mitochondria of living cells. A solubilization solution is added to dissolve the insoluble purple formazan product into a colored solution. The MTT assay was performed using a standard protocol and optical density was measured at 570 nm using a spectrophotometer.

### 3.10. Terminal Deoxynucleotidyltransferase–Mediated Biotinylated dUTP Nick End-Labeling

Terminal deoxynucleotidyl transferase–mediated dUTP nick end labeling (TUNEL) was performed by using *in situ* cell death detection kit (Roche) as previously described [6].

### 3.11. Flow Cytometry

Collected cells were incubated with AnnexinV-APC, PI, or both (BD Pharmingen) in 1× AnnexinV binding buffer (BD Pharmingen, San Diego, CA, USA) for 30 min at 4 °C in the dark and then analyzed on BD FACSA ria flow cytometer (BD Pharmingen, San Diego, CA, USA). Unstained and nontreated cells were used as control. Data were collected and analyzed with the manufacturer’s software, and Annexin V-APC(+)/PI(−) cells were gated as the apoptotic cell population.

### 3.12. Luciferase Reporter Constructs and Site-Directed Mutagenesis

The seed sequence (31–37nt) of HIF-1α 3′-UTR with flanking sequences were amplified from genomic DNA of HEK-293 cells. UTR-wt (−95 bp to +83 bp, with +1 being the first base after stop codon) was prepared with the primers HIF-1α-XbaI-P1 (5′-TCT AGA TAC AAG GCA GCA GAA AC-3′) and HIF-1α-XbaI-P2 (5′-TCT AGA GTT TGT GCA GTA TTG TAG CC-3′). PCR products were cloned into pMD18-T then subcloned into pGL3-Promoter (Promega) and designated as pGL3–UTR-wt, in which the seed sequence was inserted as the 3′-UTR downstream of the luciferase coding sequence. A construct with seed sequence in tandem (pGL3–UTR-wt) was prepared by cloning of the ligated seed sequence. Overlapping PCR was used for site-directed mutagenesis of the seed sequence (from ACACTGG to CAGATCT, designated as pGL3-UTR-mut). The PCR primers used were as follows: HIF-1α-UTR-mut1 (5′-GGC AGA TCT TGG CTC ATT ACC-3′), HIF-1α-UTR-mut2 (5′-CAA GAT CTG CCA AAA AAA GGA ATG-3′).

### 3.13. Dual Reporter Gene Assay

PC-3 cells were cultured in 24-well plates and transfected with 0.8 μg of the reporter constructs by using Lipofectamine 2000 (Invitrogen, Carlsbad, CA, USA). The pRL-CMV plasmid (Promega, Madison, WI, USA) containing the Renilla luciferase gene (0.03 μg) was cotransfected as internal control. Cells were infected with AD-miR199b or AD-control (multiplicity of infection, 100) 4 h after transfection, collected 24 h later, and the firefly and Renilla luciferase activities were assayed on Luminometer TD-20/20 (Turner Design, Fresno, CA, USA).

### 3.14. Statistical Analysis

The SPSS 18.0 program was used for general statistical analysis. All quantitative data were analyzed using Student *t*-tests. *p* < 0.05 was considered to be statistically significant.

## 4. Conclusions

In summary, this study demonstrates that miR-199b is dramatically down-regulated in prostate cancer and HIF-1α is a direct, functional target of it. Reduced miR-199b expression could contribute directly to elevated expression of HIF-1α in prostate cancer cells. Artificial overexpression of miR199b in prostate cancer cells result in decreased cell growth and increased apoptosis. Therefore, miR-199b has potential application as a biomarker or therapeutic agent for prostate cancer.
